# Subsea permafrost organic carbon stocks are large and of dominantly low reactivity

**DOI:** 10.1038/s41598-023-36471-z

**Published:** 2023-06-09

**Authors:** F. Miesner, P. P. Overduin, G. Grosse, J. Strauss, M. Langer, S. Westermann, T. Schneider von Deimling, V. Brovkin, S. Arndt

**Affiliations:** 1 Alfred Wegener Institute Helmholtz Centre for Polar and Marine Research, Potsdam, Germany; 2 Institute of Geosciences, University of Potsdam, Potsdam, Germany; 3 Department of Earth Sciences, Faculty of Sciences, Vrije Universiteit Amsterdam, Amsterdam, The Netherlands; 4 Department of Geosciences, University of Oslo, Oslo, Norway; 5 Center for Biogeochemistry in the Anthropocene, University of Oslo, Oslo, Norway; 6 Max Planck Institute for Meteorology, Hamburg, Germany; 7 CEN, University of Hamburg, Hamburg, Germany; 8 BGeoSys, Department of Geosciences, Environment and Society, Université libre de Bruxelles, Brussels, Belgium

**Keywords:** Carbon cycle, Cryospheric science

## Abstract

Subsea permafrost carbon pools below the Arctic shelf seas are a major unknown in the global carbon cycle. We combine a numerical model of sedimentation and permafrost evolution with simplified carbon turnover to estimate accumulation and microbial decomposition of organic matter on the pan-Arctic shelf over the past four glacial cycles. We find that Arctic shelf permafrost is a globally important long-term carbon sink storing 2822 (1518–4982) Pg OC, double the amount stored in lowland permafrost. Although currently thawing, prior microbial decomposition and organic matter aging limit decomposition rates to less than 48 Tg OC/yr (25–85) constraining emissions due to thaw and suggesting that the large permafrost shelf carbon pool is largely insensitive to thaw. We identify an urgent need to reduce uncertainty in rates of microbial decomposition of organic matter in cold and saline subaquatic environments. Large emissions of methane more likely derive from older and deeper sources than from organic matter in thawing permafrost.

## Introduction

The magnitude and distribution of permafrost below the Arctic shelf seas are major unknowns for permafrost organic carbon (OC) pools^1^. Sea level variations over glacial-interglacial cycles have repeatedly exposed and inundated the shelf, creating alternating terrestrial and onlapping marine strata observable in deeper sediment cores (e.g.^2^). Since the end of the Last Glacial Maximum ($\sim$ 21 thousand years ago, kyr) an estimated 4.7 $\times 10^{6} \text{ km}^2$ of terrestrial permafrost^3^ has been inundated, of which approximately 2.5 $\times 10^{6} \text{ km}^{2}$ still persists today^4^, based on numerical modelling of the thermal state of permafrost over the last four glacial cycles. The thickness of this permafrost has been constrained using industry data (e.g. boreholes^5,6^ and geophysics^7,8^) and modelling (e.g.^9^), which show permafrost reaching more than 700 m thick below the sea floor. Since its inundation, shelf permafrost temperature has adapted to marine bottom water temperatures. Subsea permafrost and its OC pool are thus in an advanced state of warming relative to cold terrestrial permafrost^10–12^. Subsea permafrost warms to its melting point within a few millenia after inundation^13^, but further warming requires melting of ice in the sediment and thawing can take several tens of thousands of years for permafrost a few hundred meters thick^14^. Although subsea permafrost is cryotic (i.e., $< {0}\,^{\circ}$C), saline sediments and the downward diffusion of saline porewater from the sea floor can lower its freezing temperature, making it free of ice (i.e., cryotic but unfrozen). Modeling studies suggest that subsea permafrost is widespread below the North American Beaufort and North Siberian shelves^4^, that it has persisted for multiple glacial–interglacial cycles^9^ but its temperature and ice content, especially in deeper waters, remain uncertain^15^.

OC burial in the sediment of the entire Arctic shelf is estimated to be 9 Tg C/yr^16–18^, providing a mechanism to sequester exagram-scale OC stocks over glacial cycles. Due to low temperatures and ice-rich sediments, permafrost slows microbial activity and thus preserves organic matter (OM), but the magnitude of permafrost’s role has not been described or estimated. How much OC is stored in subsea permafrost is therefore very poorly constrained^1,16,19^. A survey of experts estimates 170–740 Pg C stored in OM and 10–110 Pg C in trapped CH_4_^19^, but estimates linked to observational data are clearly needed to constrain uncertainty. Subsea permafrost total carbon stocks below the East Siberian Sea have been estimated at 1400 Pg C^20^ based on adding an estimated 500 Pg C in organic matter in the uppermost 25m of inundated terrestrial permafrost^21^ to 900 Pg C in greenhouse gases in free and hydrate form. Similar amounts are attributed to the entire Arctic shelf area^22,23^. The degree of decomposition of terrestrial OM derived from permafrost and deposited under marine conditions also depends on how much time it spends under oxic conditions during cross-shelf transport^24^. Once buried deeper than a few centimeters, most shelf sediments are anoxic and decomposition slows down. OM incorporated into terrestrial permafrost and subsequently inundated by sea water is not affected by cross-shelf transport, but its microbial decomposition is affected by community composition and the sediment environment. Although permafrost thus limits OM decomposition due to low temperatures and a reduction in microbial habitat as part of the sediment pore volume is replaced by ice, the depression of the freezing point by salt permits microbial activity in cryotic sediment by preserving liquid water habitat at subzero temperatures^25^. Lack of knowledge of permafrost distribution, temperature and soil composition has hampered quantification of the subsea permafrost OC sink’s longevity and vulnerability under current and future climate conditions. The poor constraint of past and future subsea permafrost distribution augments the knowledge gap and has prevented inclusion of subsea permafrost thaw effects in Earth System Models^26^, or in climate assessments such as SROCC or IPCC AR6^27,28^.

Permafrost OC stocks on land are larger than current atmospheric stocks^1^ and are linked to climate. Their future release is sensitive to carbon emission pathways^27^ and creates a positive feedback loop between climate warming and one of its consequences, permafrost thaw. Subsea permafrost has already undergone a rapid warming of about 10–15 °C due to marine transgression^4^, compared to the warming of 0.2–0.4 $^{\circ }$C per decade of continuous terrestrial permafrost^29^ in the early 21st century. Subsea permafrost warming and thaw is therefore expected to be further advanced than for terrestrial permafrost. This post-transgression warming may have created pathways for gas flow, particularly through ebullition, or have destabilized gas hydrates. Both have been invoked to explain observations of greenhouse gas release from the Arctic seabed^30^, although the source of gas remains controversial^31–34^. Field studies of carbon sources^35,36^, studies of frozen sediment^4,37^, and constraints on permafrost distribution^4,7,38^ have not led to consensus on current methane flux emissions and sources nor to an understanding of their relationship to permafrost or its thawing. There remains therefore an urgent need to better estimate OC and greenhouse gas pools associated with subsea permafrost below the Arctic shelf. In this study, we estimate the accumulation of OC in subsea permafrost and its decomposition over four glacial cycles in order to estimate modern OC stocks and to constrain potential greenhouse gas emissions due to the decomposition of this OC as permafrost thaws.

We used model results of the thermal state and the liquid pore water fraction of the subsurface over the last 450 kyr^4^ and conceptualized microbial decomposition of buried OM as a function of the liquid water habitat in the sediment. Our modeling shows that subsea permafrost has accumulated around 2800 Pg OC over the past 450 kyr, until pre-industrial time. This is approximately 2 times the estimated terrestrial permafrost region OC stock (1460–1600 Pg OC, including unfrozen deposits in that region^39–44^), although the area underlain by subsea permafrost is approximately only 18% of its terrestrial counterpart^45^. Our model results are based on all sediment deposited within the model time—up to 150m  below sea floor—while the estimate for the terrestrial OC stock is restricted to only the uppermost 50 m, independent of deposition period. Comparing the same depth intervals as a proxy for deposition period underscores the larger OC reserve on the shelf: 90% of the OC stock in subsea permafrost is located in the upper 50 m below sea floor of the sediment column (Supplementary Table S2). The persistent presence of permafrost on the Arctic shelves over the past up to 2 Myr^9^ is thus likely to have preserved more OC than is currently estimated to be stored in permafrost on land, forming a substantial long-term sink for terrestrial carbon in the Earth System.

Our work thus shows that a vast pool of OC has been accumulating beneath the Arctic Shelf over multiple glacial cycles. The reactivity of this remaining OC and the degree of its preservation from aging by permafrost, play a decisive role in determining potential emissions from microbial decomposition when it thaws. However, our model also shows that, since this OC pool is mostly aged and has been subject to decomposition, what remains is not very reactive. We model current permafrost OC decomposition rates to be 1.4 Tg C/yr (0.8–2.1) within sediments deposited over the past 450 kyr. Accelerated permafrost thaw will increases these rates, but with an upper bound of 48 Tg C/yr (25–85) based on Arctic shelf depositional history, placing an upper bound on potential emissions. We conclude that subsea permafrost OC is largely insensitive to thaw, but that a better understanding and quantification of its preservation with field data would help constrain risks associated with this first modeled estimate. The implication is that decomposition products, i.e. greenhouse gases, have been sequestered within and/or below permafrost (the former due to upward thaw at the permafrost base) in some form, or were emitted from the seabed. To better constrain climate risks associated with emissions from gas hydrate dissociation and/or greenhouse gas emissions, our understanding of subsea permafrost thaw, its overlap with gas hydrates and the microbial control on emissions of carbon dioxide vs. methane must be improved.

## Results

### Organic carbon accumulation below the Arctic shelf

**Figure 1:**
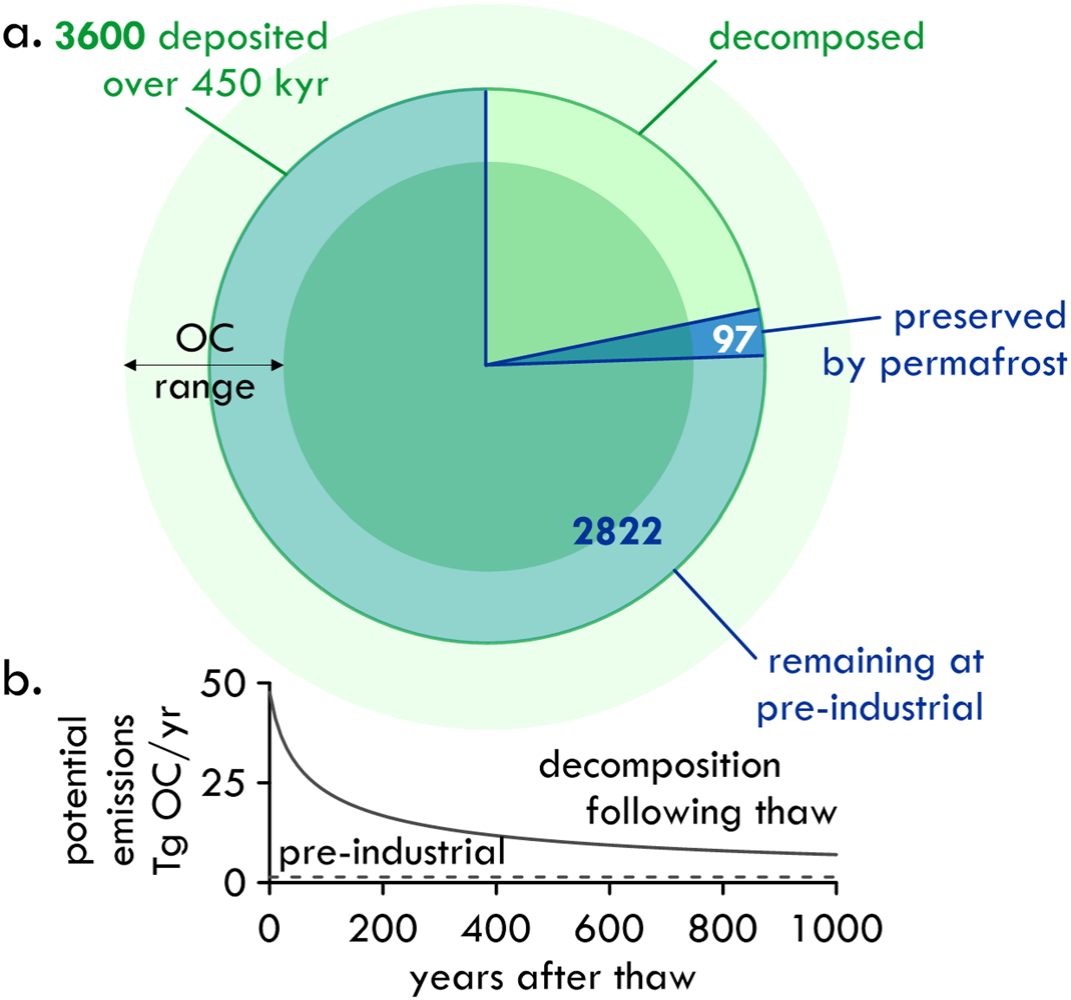
(**a**) Organic carbon stocks in subsea permafrost at pre-industrial time and potential emissions. In our model 3600 Pg C are buried on the Arctic shelf over the past 450 kyr (green circle). The radial range stems from plausible variability of OC deposition rates (Table 1). This carbon decomposes slowly over time, leaving 2822 Pg C remaining at pre-industrial time. Permafrost plays a small role in OC quantity—only 97 Pg C of these 2822 Pg C are preserved because of permafrost (blue slice), since slow decomposition occurs even under cryotic conditions. (**b**) When thawed, OM decomposition rates increase 30-fold from about 1.4 Tg C/yr (at pre-industrial time for all sediment deposited over the model period) to over 47 Tg C/yr, and drop over 1000 years to 7 Tg C/yr (lower graph). Realistic thaw rates for subsea permafrost, however, lie below 5 cm/yr over this period^10,26^ and constrain decomposition rates to below this latter value.

Buried OC is calculated in our model from spatially uniform net mass accumulation rates of terrestrial and marine material^4^ with assigned OC contents based on medians of observational data for marine and terrestrial sediments (Table 1). Sediment OC content before 450 kyr before present (BP) was ignored, i.e. we considered only OC accumulated within our modeled time period. With this simplilfied approach, we estimated that 3600 Pg C was buried beneath the seabed over the past 450 kyr for the portion of the Arctic Shelf region currently underlain by permafrost (Fig. 1a). However, both accumulation rates and OC contents vary greatly in space and time in response to glacial and deglacial cycles, with associated changes to drainage of watersheds and glacial isostatic adjustment, and in response to sea level changes. Recent work shows that deglaciations can amplify permafrost carbon release, primarily by mobilizing organic-rich surficial deposits which are then transported to the sea^46–49^. This carbon may partially correspond to sediments of today’s Yedoma region, which currently covers 2.6 $\times 10^{6}\text{ km}^{2}$ of Siberia^50^ and presumably extended far northward of the modern coastline^9,51^. To estimate the effect of variability in OC deposition, we applied 25–75% quantiles of the OC contents in fresh material for both marine and terrestrial sedimentation, generating burial of an OC range: 1960–6140 Pg C (Fig. 1a).

Previous estimates of recent OC deposition are 10 Tg C/yr for the Russian Arctic shelf (2.15 g C/$\text{m}^2$/yr)^18^ and 8.7 Tg C/yr for the entire Arctic shelf (1.73 g C/$\text{m}^2$/yr)^17^ and are of the same order of magnitude as our mean modelled OC deposition rate: 8 Tg C/yr (4.4–13.6) for the past 450 kyr over the entire modelled region and 11.9 Tg C/yr (6.8–17.2) at pre-industrial time for the subsea permafrost region. Current estimates of terrestrial OC sedimentation rates on the Arctic shelf range from 1.8 Tg OC/yr to 13 Tg OC/yr (means for shelf seas: minimum, Beaufort, to maximum, East Siberian)^52^ based on sediment OC contents and Pb-dating of sediments for accumulation rates, for a total of 45 Tg OC/yr for the entire Arctic Shelf. The burial efficiency is estimated at around 10%^18^, which would imply accumulation of around 4 Tg OC/yr, but this estimate excludes marine derived OC. As a function of sediment depth, our OC contents (${1.16}\;{wt\%} \pm 0.75$) fall within the range of observed values from shelf boreholes (mean ${1.06}\;{wt\%} \pm 1.05$, Supplementary Fig. S1c,d^53–56^). Our sedimentation scheme thus generates initial shelf OC stocks that correspond to observationally based estimates of burial rates.

### Decomposition of organic matter in subsea permafrost

We calculated OM decomposition rates with a reactivity continuum model (RCM) (Refs.^57,58^, “Methods” section), exploring subsea permafrost OC evolution for a plausible range of reactivity parameters. We chose different parameter sets for marine and terrestrial deposits based on measurements^58,59^ and the inversion of observed incubations from the Siberian Shelf^37^. Output from our thermal model included sediment temperature and its liquid water and ice contents^4^, so that both temperature and the available volume of microbial habitat could be used as limiting factors for microbial activity.

To address uncertainties in decomposition response to temperature, we performed two simulations. In the first simulation, we only relate decomposition to the availability of liquid water in the sediment pore space, regardless of sediment temperature (Eq. (5), “Methods” section). In this case, slow decomposition occurs even under cryotic conditions and OC stocks of approximately 2822 Pg C (1518–4982, for quartiles of initial OC content) remained at pre-industrial time (Supplementary Table S1), i.e. approximately 22% (19–29) of the deposited OM had decomposed over the 450 kyr period (Fig. 1a).

In the second, more conservative reactivity simulation, in which decomposition was halted completely in cryotic sediment (Eq. 4, “Methods” section), 3252 Pg C (1762–5633) remained and only 10% (8–10) of the deposited OM had decomposed.

**Figure 2:**
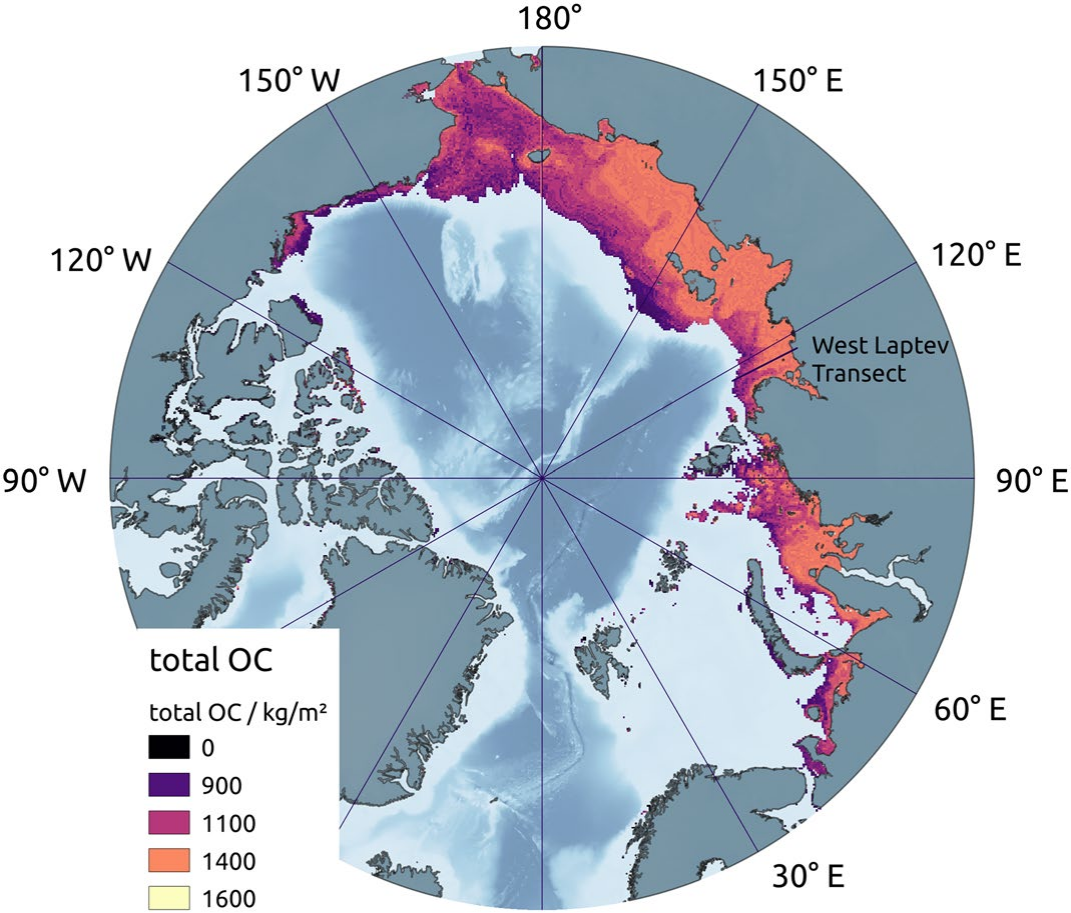
Total organic carbon content within subsea permafrost at pre-industrial time. The map shows the total amount of OC, summed over the sediment that accumulated within the last 450 kyr. OM decomposition was permitted in the liquid water volume of the sediment column, even at cryotic temperatures, with median terrestrial and marine sediment OC burial rates and OM reactivity parameters of $a={50}\,\text{yr}$ and $\nu = 0.15$ for marine and $a={0.25}\,\text{yr}$ and $\nu = 0.003$ for terrestrial deposits. Locations where subsea permafrost was not present at pre-industrial time are excluded.

We found the highest amounts of OC ($> {30} \text{{ kg m}}^{-3}$) in the regions that spent the most time under subaerial conditions, i.e., in coastal and never-glaciated regions, which results in total column OC accumulations of up to 1400 kg m^−2^ over the last 450 kyr in central and near-shore shelf areas (Fig. 2). These regions had the highest proportion of terrestrial sediment, which contained more OC than marine-dominated sediments further out on the shelf (Table 1), and had deeper and colder subsea permafrost^4^, so that OM decomposed more slowly than further offshore.

**Table 1:** Stratigraphy divisions and their OC contents.

Stratigraphy	Sedimentation	OC contents		
	Rates	1st quartile	Median	3rd quartile
Marine	30 cm/kyr (0.3 mm/a)	0.57 wt%	0.99 wt%	1.43 wt%
Terrestrial	10 cm/kyr (0.1 mm/a)	1.02 wt%	1.90 wt%	3.40 wt%

Sedimentation rates are assumed as in^4^. OC contents are taken from^43,53,60^. Quartiles are defined using the distribution of observations shown in Supplementary Fig. S1a,b.

### Preservation of organic matter by subsea permafrost

To assess the impact that permafrost has on OM preservation we compared our OC accumulation results to a scenario in which we allowed decomposition to occur independently of temperature, equivalent to a permafrost-free shelf scenario (Supplementary Table S1). The percentage of post-industrial permafrost OC that results solely from the presence of permafrost over the last 450 kyr is a measure for the permafrost preservation effect.

**Figure 3:**
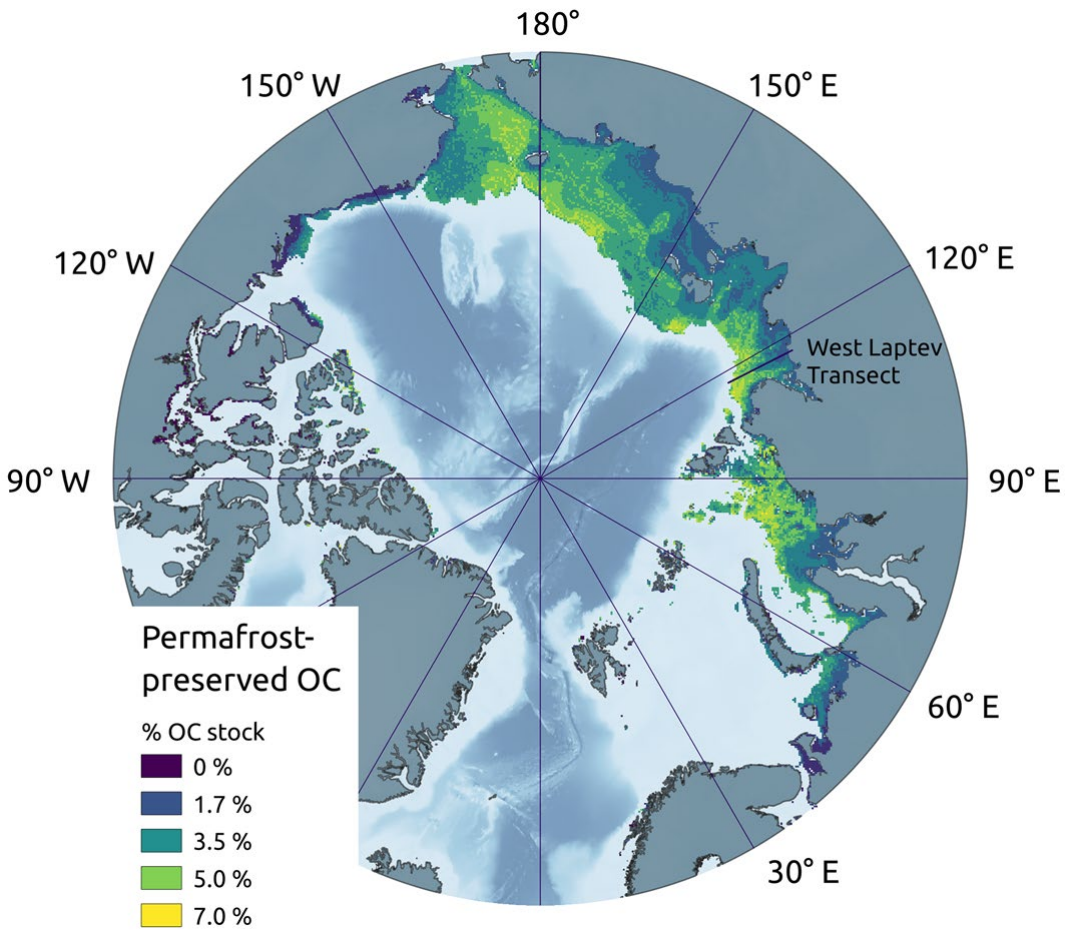
Percentage of organic carbon content that was preserved by the presence of permafrost during the last 450 kyr in the region underlain by subsea permafrost at pre-industrial time. The color scale indicates the percentage of pre-industrial OC stock (Fig. 2) preserved by permafrost (i.e. % OC exceeding OC accumulation in the absence of permafrost).

We found that only marginally more OC is stored with permafrost (2822 PgC) than without (2725 PgC), a preservation effect of 3% (Fig. 3). For the case in which decomposition occurs whenever sediment is above 0 °C, the preservation effect is higher at 16%. Magnitudes of preservation reached a maximum of around 60 Tg $\text{ km}^{-2}$ but were spatially variable (Fig. 3). Applying terrestrial-fit reactivity parameters to the whole sediment column, the preservation effect is even lower ($< {1.2}\%$). Higher reactivity parameters, corresponding to marine OC reactivity, result in a preservation effect of 48%, i.e. permafrost almost doubles OC storage when reactivity is high but has little effect when the OC is difficult to decompose (Supplementary Table S1). As a result, permafrost typically preserves a smaller fraction of OC close to the present-day coast (where terrestrial sediment dominates) than it does midshelf, particularly below the broad shelves of the East Siberian Sea (Fig. 3). In areas with high marine fraction in the sediment column, locally up to 7% of the OC remaining after 450 kyr was preserved by the presence of permafrost on the largely unglaciated shelves of the eastern Kara, the Laptev, the East Siberian and the Chukchi seas.

**Figure 4:**
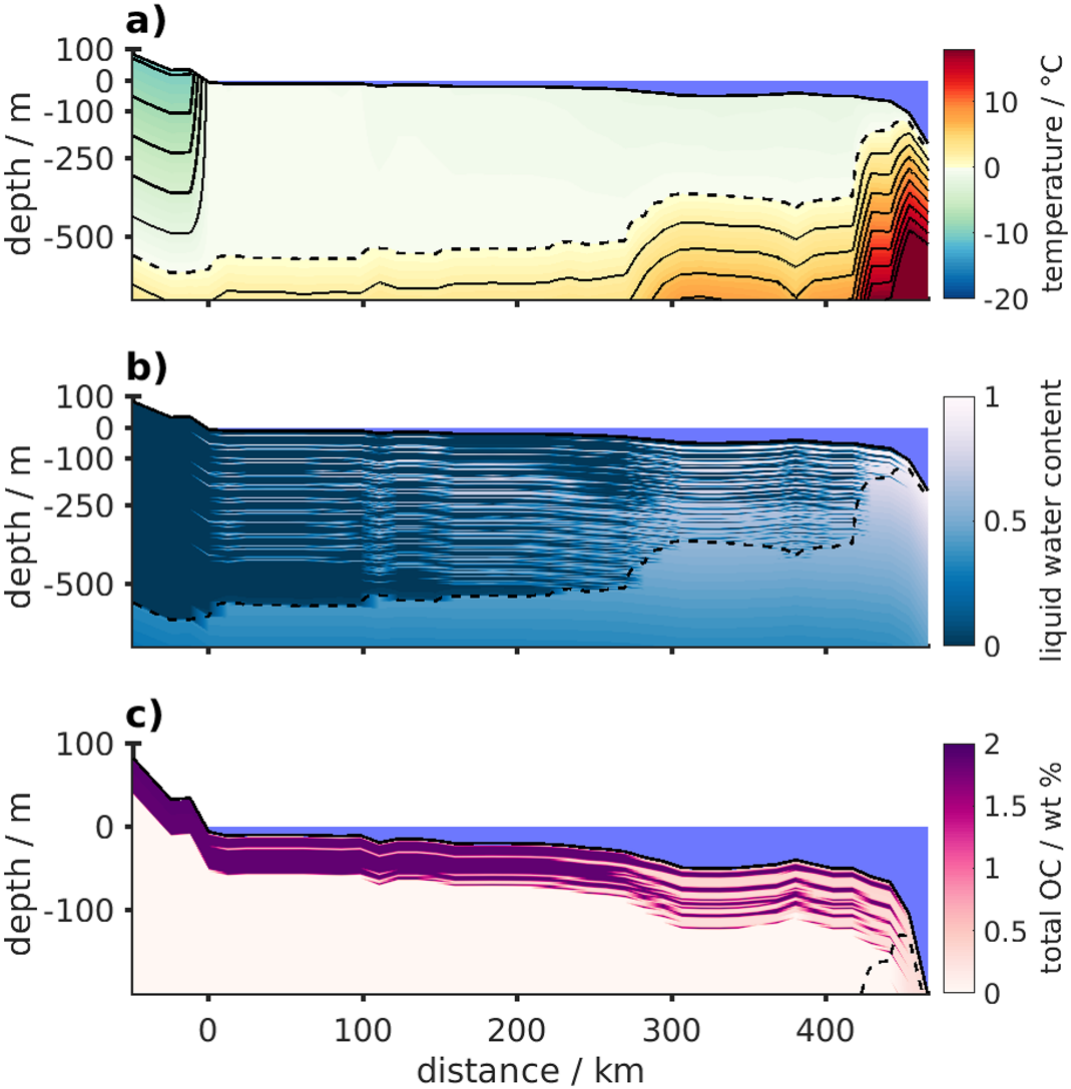
Organic carbon degradation illustrated on a south-north transect in the western Laptev Sea. Sediment temperature (**a**), liquid water content in the pore space (**b**), and organic carbon content (**c**) stored along a south-north transect (from left to right) in the western Laptev Sea (along 117° E) at pre-industrial time. Panel (**c**) shows sediment organic carbon (OC in wt%) for decomposition scaled to liquid water content. OM was only accumulated within the last 450 kyr with median terrestrial and marine sediment OC burial rates and decomposition was scaled with reactivity parameters of $a={50}\,\text{yr}$ and $\nu = 0.15$ for marine and $a={0.25}\,\text{yr}$ and $\nu = 0.003$ for terrestrial deposits. The location of this transect in the western Laptev Sea is shown in Figs. 2, 3. Note the different depth scales between panels (**a**)/(**b**) and panel (**c**).

These patterns of how OM preservation varies with sediment and OM reactivity are illustrated in Fig. 4. The total OC content at pre-industrial time is shown for a north-south depth profile with approx. 600 m thick permafrost at the coastline of the western Laptev Sea (Ref.^4^, transect location indicated in Figs. 2, 3), where large differences in the OC content can be observed between the different layers of sediment: as a result of both higher reactivity and higher liquid water content at temperatures below 0 °C, more OC is stored in non-saline terrestrial sediment layers than in saline onlapping transgressive sediments (Fig. 4c).

### Decomposition rates after thaw

To assess the effects of present and future permafrost thaw, we additionally modelled the OM decomposition rates beginning from pre-industrial time (1850CE) until 3000CE, permitting OM to age regardless of temperature or ice content. This is equivalent to permitting OM reactivity to vary as it would in the absence of permafrost, in either a cryotic ($\le {0}\,^{\circ }$C) or thawed (unfrozen) sediment, respectively. It simulates the instantaneous thaw at 1850CE of sediment and OM that had been deposited over the 450 kyr before pre-industrial time and is therefore an upper bound on greenhouse gas production due to thaw of modern subsea permafrost.

Within the subsea permafrost sediment volume beneath the entire Arctic shelf, this instant thawing increased OC decomposition rates from 1.4 Tg OC/yr (0.8–2.1 Tg OC/yr) at the end of our model time to 48 Tg OC/yr (25–85 Tg OC/yr), a 34-fold increase. Rates of subsea permafrost thaw are, however, low, since mean annual seabed temperatures for much of the Arctic shelf are negative^61^. Inferred subsea permafrost thaw rates range from tens of cm/yr following transgression to less than a cm/yr after a millennium of warming beneath the seabed^10^. Modelled thaw of subsea permafrost under future warming scenarios ranges from 1–5 cm/yr over the period from today until 3000CE^26^, the latter under the high-emission SSP 8.5 scenario^27^. Earth system modeling suggests that future thaw of subsea permafrost can be directly linked to a reduction of sea ice cover^26^, but that complete thaw of subsea permafrost is unlikely to occur by 3000CE. Rates of OC decomposition are thus likely to be at least three orders of magnitude lower than our upper bound of 48 Tg OC/yr. Even in the case of complete thaw of the sediment deposited in our model runs, prior decomposition of the OM will have decreased decomposition rates by more than a factor of 6 after 1000 yr, to less than 8 Tg C/yr (Fig. 1b).

## Discussion

Although our model generates a globally significant pre-industrial OC pool beneath the Arctic Shelf, it probably underestimates this pool for a number of reasons. Firstly, our estimate of Arctic shelf OC stocks does not include OC decomposition products that may be stabilized or trapped by permafrost (e.g., gas hydrates^38^). Since gas diffusivity in frozen permafrost is orders of magnitude lower than in unfrozen sediment, ice-bearing permafrost slows or effectively halts gas diffusion^62^. Permafrost can thus prevent upward greenhouse gas migration, capping gaseous C stocks and delaying their re–introduction into the global carbon cycle^63^.

Gas hydrates within or below permafrost (permafrost-associated) may derive from underlying reservoirs, which produce thermogenic gas that migrates to shallower depths, where it may integrates into permafrost and is stabilized by lower temperatures^64^. Estimates for the amount of gas hydrates associated with permafrost in the Arctic range from around 1% of global gas hydrates (20 Pg)^38^ to estimates that suggest up to 800 Pg of methane may have accumulated below and within permafrost in the period of one glacial cycle^31^. Inclusion of gas hydrates in thermal properties of the permafrost would improve the model but would be spurious given our poor knowledge of gas hydrate distribution for most of the Arctic shelf. The presence of gas hydrates lowers the thermal conductivity of the permafrost sediments^65^. Its latent heat of formation or dissolution can similarly increase heat transfer requirements^66^ increasing thermal inertia. Gas hydrate dissolution, usually via pressure changes associated with thaw from above or via geothermal warming, can change the pore space morphology of the sediment and freshen pore space fluid^67^, raising its freezing point. Because the gas hydrate stability zone is different from the stability field of liquid water, permafrost-associated gas hydrates can exist at depths that overlap (intrapermafrost) and extend below (subpermafrost) permafrost. Understanding where gas hydrates reside in the sediments is therefore an important factor to fully account for thermal dynamics in future submarine permafrost development.

Groundwater flow and associated changes in sediment salinity can also lead to changes in permafrost distribution, for example through thaw^68^ or an increase in its extent^69^. Analogous to gas hydrates, our ignorance of ground water flow makes its inclusion in thermal modeling speculative. The effect of including ground water would change permafrost distribution but have little effect on sediment OM decomposition for the reasons described above; it would increase OC stocks and potential emissions without recourse to OM decomposition. Finally, OM decomposition may be inhibited by end product accumulation, including CO_2_ and CH_4_, which are confined almost everywhere by low gas diffusivity of the frozen sediment, and by the low solubility of saline porewater solutions.

A second factor that probably renders our estimate of the subsea permafrost OC pool too low is the choice of moderate parameters for the reactive continuum model of decomposition. Although the chemical composition of OM has traditionally been regarded as exerting the dominant control on its reactivity, recent studies support the notion of apparent OM reactivity as a dynamic ecosystem property (e.g.^70–73^). In this case, reactivity is controlled by the chemical composition of the OM, and also by a complex and dynamic interplay with environmental variables such as temperature, microbial community structure and abundance, bio-energetics, enzyme kinetics or protection by mineral surfaces^74–76^. The parameters of OM decomposition models implicitly account for these controls and, thus, vary widely across different environments^58,59^. We used two sets of values for transgressive and regressive sediment layers over the whole model area and time based on a compilation of inversely determined values from measurements and incubation experiments (see “Methods” section). The comparably high apparent reactivity of marine OM leads to faster decomposition than the lower apparent reactivity of OM stored in terrestrial deposits, where we see a high initial apparent reactivity followed by a near stop in decomposition. Using the marine OM reactivity parameters for the whole sediment column resulted in an overall smaller OC pool (1985 Pg C), but with greater permafrost preservation effect (933 Pg C).

## Methods

### Permafrost model

The thermal state of the subsurface was estimated with a 1D numerical model [Cryogrid2^77^] solving the heat equation over a 7000 $\times$ 7000 $\text{km}^{2}$ grid^4^ of 12.5 km spacing. Sedimentation and permafrost dynamics were modelled over the last four glacial cycles (last 450 kyr) on the Arctic shelf between −120 and 18 m above modern sea level, an area of approximately 4.5 $\times 10^{6}{\text{ km}^{2}}$ and therefore roughly twice the area of modern subsea permafrost^4^. The thickness of sedimentary deposits was taken from the GlobSed database^78^, below which bedrock was modelled. For each grid cell the model produced sediment temperature, porewater salinity, and volumetric ice and liquid water contents within 2 km below the seafloor or land surface at 2 m discretization over the last 450 kyr.

The model run was initialized with the steady state solution between the local geothermal heat flux interpolated from the global database of^79^ and the surface condition at 450 kyr BP for all modelled locations. We synthesized the dynamic surface forcing condition from sea level history, glaciation and time series of mean annual air temperature over the last 450 kyr using the sea level curve from^80^, and glacial ice cover extent and thickness and surface air temperature simulated by the intermediate complexity Earth System Model CLIMBER-2^81,82^.

### Organic carbon in shelf sediments

Organic carbon contents of sediment and linear net sedimentation rates were set for terrestrial and marine deposition for the circumarctic model region. To initialize the OC pool of subsea permafrost, we linked estimated OC contents to the modelled sediment stratigraphy. Available observations of total organic carbon contents for terrestrial Weichselian deposits and for Arctic shelf marine sediments are shown in Supplementary Fig. S1a–c. Observed sediment OC contents span a wide range of values. To reflect this variability as a source of uncertainty in our model, we also calculated stocks for the first and third quartile OC contents based on these observational data sets.

**Figure 5:**
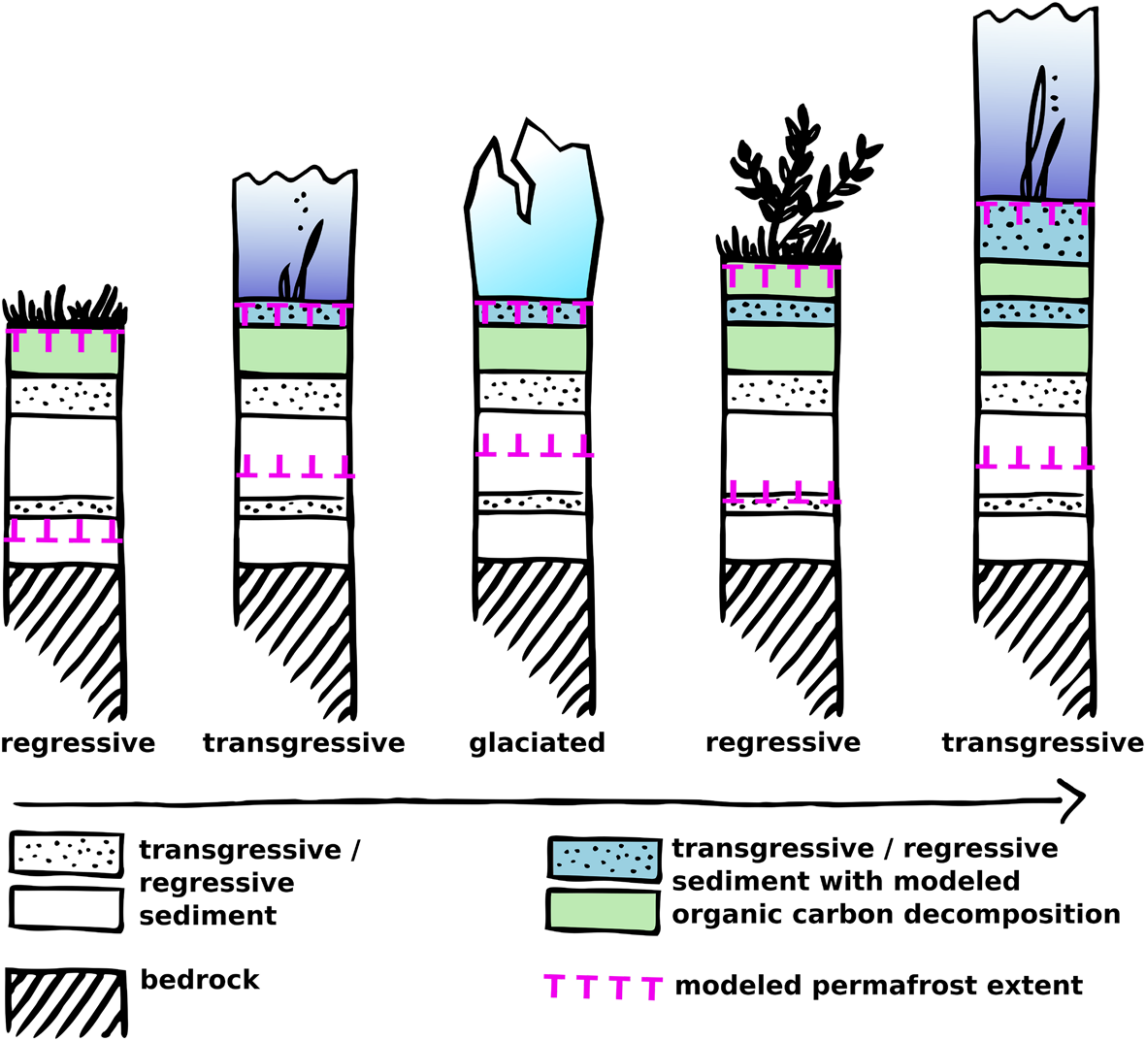
Modeling of sedimentation and heat flow during regressive (first and fourth column), transgressive (second and last column) and glaciated (third column) periods produced a simplified sediment stratigraphy and permafrost extent below the sea floor (dashed magenta lines) over depth. Sedimentation, OM contents and decomposition rates were modelled for the Arctic shelf for the past 450 kyr (time arrow). OC stocks are reported for sediment that accumulated over this period (colored strata).

We modelled deposition over the last 450 kyr at the linear net sedimentation rates chosen in Table 1, resulting in deposition of 45–135 m of the upper sediment column. For these sediment layers, OC decomposition began at the time of deposition with initial contents of fresh organic matter depending on the depositional milieu (i.e. regressive/transgressive). For deeper (i.e. older than 450 kyr) material, the carbon content was initialized as zero, equivalent to the absence of any OC in deeper sediment strata (Fig. 5).

### Organic matter decomposition

OC content over time was estimated based on time elapsed since deposition and on the sediment’s physical state. To calculate the OC content of sediment buried for time *t*, where $t=0$ is the time of sedimentation, we employed the reactive continuum model^57,58^ for organic decomposition

1 $$\begin{aligned} \text {OC}(t) = \text {OC}_{0} \left( \frac{a}{a + t} \right) ^{\nu }, \end{aligned}$$

with initial values $\text {OC}_{0}$ based on Table 1.

As a first-order estimate, we used a *decomposition time* $\tau$ in Eq. (1), that disregards time periods where the sediment’s temperature is below 0 °C, preserving OC reactivity until positive temperatures are restored. With the indicator function

2 $$\begin{aligned} \chi (t) = {\left\{ \begin{array}{ll} 0 &{} \quad T(t) < {0}^{\circ}{\text{C}} \\ 1 &{} \quad T(t) \ge {0}^{\circ}{\text{C}} \\ \end{array}\right. }, \end{aligned}$$

we defined the decomposition period as

3 $$\begin{aligned} \tau (t) = \int _{0}^t \chi (s) \, ds \end{aligned}$$

and Eq. (1) becomes

4 $$\begin{aligned} \text {OC}(t) = \text {OC}_{0} \left( \frac{a}{a + \tau (t)} \right) ^{\nu }. \end{aligned}$$

In saline marine sediments, the 0$^{\circ }$ C isotherm and the frozen/unfrozen interface are usually not coincident due to freezing point depression of the brine in the pore water. We therefore additionally linked the microbial activity to liquid water content rather than to temperature alone. Assuming that microbial activity is limited to the volume of sediment liquid water content $\theta$, we adjusted the decomposition period by the volume fraction of liquid water to

5 $$\begin{aligned} \tau (t) = \int _{0}^t \frac{\theta (s)}{\eta } \, ds, \end{aligned}$$

where $\eta$ is the porosity. The fraction $\frac{\theta (s)}{\eta }$ yields a value between zero and one. The effective decomposition time was therefore linearly scaled to the available liquid water habitat in the sediment. This ignores the effects of differences in temperature or salinity on reactivity, equivalent to the assumption that OM decomposition is primarily dependent on habitat availability, and that halocryophilic communities are adapted to a range of environmental conditions. We understand that this introduces currently unquantifiable uncertainties.

### RCM parameterization

The reactivity parameters *a* and $\nu$ of the RCM are essentially free, positive parameters that completely determine the shape of the initial, continuous distribution of OM compounds over the reactivity spectrum and, thus, the apparent reactivity of OM and its evolution during burial in the sediment^57^. In general, low *a* and high $\nu$ values indicate a dominance of more reactive compounds and thus result in a higher apparent OM reactivity, while high *a* and low $\nu$ values indicate the dominance of less reactive compounds and a low reactivity^58^. In addition, the value of *a* also controls the lifetime of the most reactive compounds and determines the decrease of OM reactivity with burial time/depth. It is often considered as an indicator for the “freshness” or “initial age” of the deposited OM^57,83^. The shaping parameters of the RCM may be inverted from incubation experiments or estimated from borehole OC records with sufficiently good age control^58,59^. Low *a* values result in a higher initial OM reactivity (for the same $\nu$) and a rapid decrease in this reactivity with OM age, while high *a* values lead to a lower initial reactivity but a slower decrease in reactivity with age (Ref.^58^, Supplementary Fig. S2). A compilation of previously published, inversely determined parameter values for marine sediments reveals that the global-scale variability in benthic OM reactivity is mainly driven by the large variability in parameter *a*. The wide range of values spans a very wide range of conditions from fresh pure marine diatom OC degrading in the lab to pre-degraded OC from marine sediments typically found on the continental slope. Published values vary from $a = {3.1e^{-4}}\,{\text{yr}}$ for highly reactive, fresh phytoplankton material that was decomposed under laboratory conditions to $a = {1.4e^{5}}\,{\text{yr}}$ for OM deposited in the Central Equatorial Pacific^57,84^. The typical value range for OC found in coastal marine sediments, however, is $a \approx$ 5 to 70 yr^58,59,85^. In contrast, reported $\nu$ parameter values for marine sediment mostly fall within the range $0.1< \nu < 0.2$^57–59^, thus indicating that $\nu$ remains relatively constant across largely different depositional environments. This finding is also fully consistent with the empirically derived power law description of OM decomposition^83^ that applies a globally constant parameter. Based on these findings, we chose $a = {50}\,{\text{yr}}$ and $\nu = 0.15$^58,59^ for the marine sediment layers in our stratigraphy.

Values of *a* and $\nu$ for terrestrial layers were inversely determined by fitting the RCM to reported ${\text{CO}}_2$ production rates during anoxic incubations of permafrost samples at $4\,^{\circ }$C reported by^37^, which are, to our knowledge, the only values available for subsea permafrost sediment OC. Best fit ($a,\nu$) couples are found by solving the least squares minimization problem:

$$\begin{aligned} min_{a,v}\sum _{i=1}^{n}\left( \frac{v}{a+t} G_0(\frac{a}{a+t})^2-R_{i,obs}) \right) ^2, \end{aligned}$$

where $R_{i,obs}$ are the measurements, $G_0$ the initial OM content and *t* denotes the incubation time. This results in values of $a = {0.0007}\,{\text{yr}}$ (1e^−8^–2e^−3^) and $\nu = 0.005$ (4e^−4^–2e^−3^). We chose $a = {0.25}\,{\text{yr}}$ and $\nu = 0.003$ for terrestrial sediment layers. Choosing *a* lower than 1 yr and a comparably low $\nu$ is fully consistent with three-pool model based on a circumpolar database for terrestrial OC that shows widely observed slow and continuous decrease in OC reactivity over time^86^.

### Sources of uncertainty

Sources of uncertainty in the reported OC stock estimates are the OC content of buried terrestrial and marine sediment on the shelf and the choice of RCM reactivity parameters *a* and $\nu$. Shelf deposits are more difficult to characterize than terrestrial, where surficial geomorphology, remote sensing and denser sampling can be used to characterize the distribution of OC stocks. Observation of shelf sediment, particularly by sampling in the shallow waters ($< {20}\,{\text{m}}$) of much of the Siberian shelf, has been limited by ice cover and restricted access to shallow-draught ships. For this reason, upscaling of local observations and propagation of observation-based uncertainties, as for OC stock estimation on land^39^, are not possible. Our OC contents are, however, based on databases of observed values of buried terrestrial Late Pleistocene and marine sediment OC^44,53,60^, which are applied uniformly over the Arctic shelf depending on whether the shelf is exposed during transgressions or inundated during regressions, respectively, to create site specific sediment stratigraphy. Variability in OC content (see Supplementary Fig. S1a,b) is characterized using the first and third quartiles (Q1, Q3) of the distributions. This approach ignores spatial and temporal heterogeneity in sedimentation and erosion but brackets results of simulations, for which we report resulting OC stocks resulting from *median* (*Q1*–*Q3*) OC contents. Holocene sediments with higher OC contents, including thermokarst lake and alas deposits^87^, peatlands^40^ or the Yedoma complex deposits^44^ are generally eroded during coastline retreat driven by thermoerosion and sea level rise along the Arctic coastline. The OM released by erosion is subsequently transported out of the shelf region (for example, by ice-rafting), subject to turnover in the water column, or deposited on the seabed, where it undergoes turnover prior to burial. Available buried OC contents on the Arctic shelf for samples of millennial to glacial cycle time-scale sediment records have lower median values than either the marine sediment or terrestrial permafrost data we use as input (Supplementary Fig. S1c,d).

Our somewhat arbitrary choice of the poorly constrained RCM parameter *a* introduces further uncertainty, and reflects a true uncertainty in our understanding of subsea permafrost OM decomposition. Low terrestrial OM reactivity make changes to parameterized reactivity negligible in terms of their impact on remaining OC stocks over long time periods. Changing *a* in sediment of terrestrial origin from our value of 0.25 to 0.0007 (from^37^), while holding other parameters equal, reduces estimated OC stocks by around 2% (from 2822 to 2778 Pg OC). Changing *a* for marine OM can affect remaining stock more dramatically, but the OC content of marine sediment can dampen the effect on total estimates. Decreasing (increasing) marine *a* while holding other parameters constant from 50 to 5 yr (500 yr) changes the total subsea permafrost OC stock estimate from 2822 to 2710 Pg OC (2980 yr), a reduction (increase) of 4% (6%), respectively.

Most incubation experiments of microbial activity in permafrost are conducted at 4-20 °C. Subsea permafrost is mostly between −2 and 0 °C where temperature variations are not as pronounced as changes in the ice content. We therefore lack data to properly parameterize the dependence of OM reactivity on temperature for subsea permafrost; however, the inversely determined parameter fits implicitly account for temperature. To account for uncertainties in our assumed parameterization, (*a*, $\nu$) couples for marine and terrestrial sediment OC were also applied to the whole sediment column for a high and a low reactivity scenario, respectively.

Two general points can be made about the uncertainties calculated as described. Firstly, uncertainty in OC stocks that results from changes to the estimates of OC content in sediment are greater than uncertainty resulting from assumptions regarding OC reactivity. The choice of different RCM parameter couples (*a*, $\nu$) results in a smaller range in the estimated OC pool, than changing the initial OC content. As an example, our most plausible estimate for remaining OC stocks, 2822 Pg OC has associated uncertainty ranges of (1518–4982) due to OC sediment content uncertainty but only (1985–3497) when making the assumption that ALL sediment is of marine or terrestrial origin in terms of its reactivity (combined, the two effects result in a range of 2822 Pg OC (1075–5963)). This suggests that constraining subsea permafrost OC stocks is more effectively done by better describing sedimentation composition than by improving our estimates of OC reactivity. Secondly, given the glacial cycle time scale for degradation, the form of the RCM itself imposes limits on the degree of decomposition that can be attained or avoided. For example, even lower *a* values will make almost no change to resulting OC stocks, since they imply such a low reactivity that much longer time scales would be required to change OC stocks appreciably.

## Supplementary Information

Supplementary Information.

## Data Availability

Input data for modelling is available from the sources listed. Output data for the permafrost map is available^88^.

## Code availability

Program code is based on CryoGrid, as described in^4^. A community version of this code is available at (https://github.com/CryoGrid/CryoGrid). The specific code for this experiment is available from the authors per request.
