# Supplementary material for: Subsea permafrost organic carbon stocks are large and of dominantly low reactivity
Source: Sci Rep. 2023 Jun 9;13:9425. doi: 10.1038/s41598-023-36471-z (PMC10256719; doi:10.1038/s41598-023-36471-z)
Supplement: Supplementary file 1 — Supplementary Information. [file 41598_2023_36471_MOESM1_ESM.pdf]

Supplementary Online Material to: Subsea permafrost organic carbon stocks are large and of dominantly low reactivity

F. Miesner[*,1], P. P. Overduin[1], G. Grosse[1,2], J. Strauss[1], M. Langer[3,1], S. Westermann[4,5], T. Schneider von Deimling[1,3], V. Brovkin[6,7], and S. Arndt[8]

[1]Alfred Wegener Institute Helmholtz Centre for Polar and Marine Research, Potsdam, Germany
[2]Institute of Geosciences, University of Potsdam, Potsdam, Germany
[3]Department of Earth Sciences, Faculty of Sciences, Vrije Universiteit Amsterdam, Amsterdam, Netherlands
[4]Department of Geosciences, University of Oslo, Oslo, Norway
[5]Center for Biogeochemistry in the Anthropocene, University of Oslo, Norway
[6]Max Planck Institute for Meteorology, Hamburg, Germany
[7]CEN, University of Hamburg, Hamburg, Germany
[8]BGeoSys, Department of Geosciences, Environment and Society, Université libre de Bruxelles, Brussels, Belgium

June 8, 2023

* Corresponding author: frederieke.miesner@awi.de

Table S1: **Resulting organic carbon that is still stored within permafrost at pre-industrial time for initialization as zero for sediment buried prior to** −450 kyr**.** The columns show the different decomposition scenarios, where degradation is permitted independent of permafrost (first column), controlled by the liquid water habitat (second column) or completely halted in cryotic sediments (third column). The first and third row represent marine- or terrestrial-fit decomposition parameters, respectively, for the whole sediment column, while the second row represents marine- and terrestrial-fit parameters assigned to their respective layers. The uncertainties in the amount of OC resulting from each scenario accounts for the uncertainty in the initial OC contents (main manuscript, Tab. 1).

**Reactivity Parameter**				**TOC** (Pg)		
marine deposits		terrestrial deposits		**no permafrost**	**permafrost**	
				(constant decomposition)	(liquid water controlled)	(temperature controlled)
a	ν	a	ν			
50	0.15	50	0.15	1052 (576–1775)	1985 (1075–3450)	2985 (1620–5142)
50	0.15	0.25	0.003	2725 (1464–4827)	2822 (1518–4982)	3252 (1762–5633)
0.25	0.003	0.25	0.003	3455 (1883–5888)	3497 (1905–5963)	3564 (1941–6079)
0.0007	0.002	0.0007	0.002	3462 (1886–5899)	3489 (1901–5949)	3562 (1940–6076)

Table S2: **Comparison of terrestrial and marine OC stock estimates.** The columns show estimates of terrestrial and marine OC stocks in different depth ranges with associated uncertainties. The marine estimates are results of this study for the liquid water habitat controlled decomposition setting and the uncertainties account for initial OC contents (main manuscript, Tab. 1). The terrestrial estimates were compiled in [42].

**Land Permafrost**			**Subsea Permafrost**	
domain	estimate / Pg	reference	domain	estimate / Pg
upper 3 m frozen and unfrozen	1034	[39]	upper 3 mbsf	93 (51 –150)
3 m to 50 m	420	[39, 40, 43]	3 mbsf to 50 mbsf	2437 (1309 - 4330)
total permafrost region	1538 (1460 –1600)	[42, 43]	total modeled subsea permafrost	2822 (1518–4982)

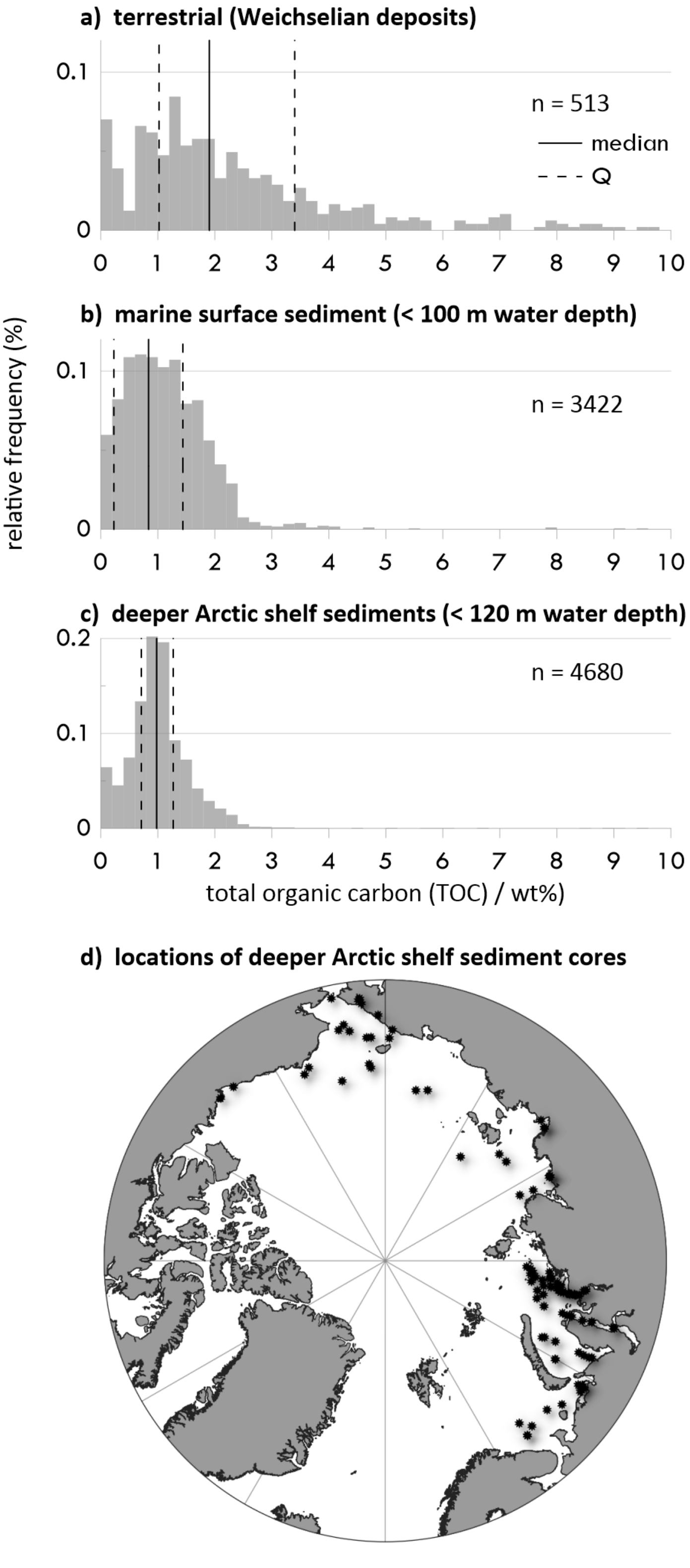

Figure S1: **Distribution of observed organic carbon (OC) contents (in** wt%**) for terrestrial sediments and marine surface sediments in the Arctic.** a) Terrestrial observations (**A**) are based mostly on analyses of coastal exposures and sediment core data from Eastern Siberia [60]. b) Marine observations (**B**) are from water depths less than 100 m, based on surface sediment analyses in the CircumArctic Shelf Sediment Carbon Database (CASCADE, [53]). c) Observed buried OC contents from available deeper boreholes ([53, 54, 55, 56]). d) Map of sediment core locations for data in histogram c).

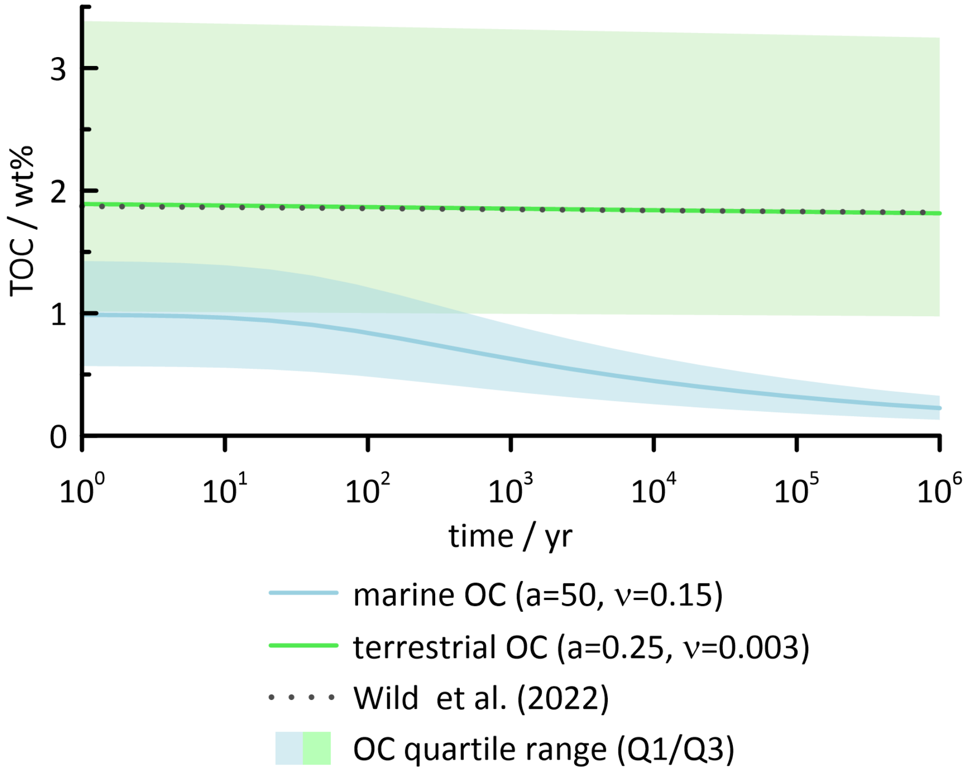

Figure S2: **Decrease in OC as a function of time as given by the reactive continuum model.** The graph shows the OC decomposition based on the simulation of liquid water microbial habitat with initial median terrestrial and marine sediment OC content (1.90 % and 0.99 %, respectively; Fig. S1) and reactivity continuum model parameterizations (a, ν) of marine: (50, 0.15) and terrestrial: (0.25, 0.003). For comparison, the OC decomposition for mean inversely determined a and ν values from incubations of subsea permafrost sediment samples from the Siberian Shelf [37]. The shaded regions indicate the range between 1st and 3rd quartiles for sediment OC content.
